# 3D transesophageal echocardiographic visualization of the pulmonary artery catheter through the tricuspid valve and their position relative to the tricuspid valve leaflets

**DOI:** 10.1186/s40981-024-00718-z

**Published:** 2024-05-30

**Authors:** Shoko Takada, Tomoko Fujimoto, Akiko Tomita-Kobayashi, Yukio Hayashi

**Affiliations:** 1 Anesthesiology Service, Sakurabashi-Watanabe Hospital, 2-4-32 Umeda, Kita-ku, Osaka, 530-0001 Japan; 2 Present Address: Anesthesiology Service, Yoka Municipal Hospital, 1878-1, Yoka, Yoka-cho, Yabu, Hyogo 667-8555 Japan

**Keywords:** Pulmonary artery catheter, 3-Dimensional transesophageal echocardiography, Tricuspid valve

## Abstract

**Background:**

The tricuspid valve is an atrioventricular valve consisting of three lobes. We used the 3D transesophageal echocardiography to visualize position of the pulmonary artery catheter at the tricuspid valve annulus and examined where the catheter passed through at the level of the tricuspid annulus.

**Methods:**

In this prospective and observational study, we monitored the pressure wave on patients undergoing cardiac surgery with the catheter placement by monitoring the pressure waveform for 8 months. We measured the time required for the catheter to pass through the tricuspid and pulmonary valves, respectively. We acquired the 3D image of the tricuspid valve by transesophageal echocardiography and determined the position of the pulmonary artery catheter at the level of the tricuspid annulus. The data were analyzed by Kruskal-Wallis test followed by Mann-Whitney test with Holm multiple comparisons. *P* < 0.05 was considered significant.

**Results:**

Of the 116 cases, the pulmonary artery catheter passed through the tricuspid valve between antero-posterior leaflets in 78 cases (67.2 %), between septal-posterior leaflets in 25 cases (21.6 %), and between antero-septal leaflets in 2 cases (1.7 %) and the center in 11 cases (9.5 %), respectively. The time required for the catheter to pass through the pulmonary valves was significantly different among the catheter positions at the level of the tricuspid annulus.

**Conclusion:**

The pulmonary artery catheter location at the level of the tricuspid annulus can be identified using 3D transesophageal echocardiography. The location of the catheter significantly affects the pulmonary artery catheter placement time.

## Introduction

The pulmonary artery catheter (PAC) is used for perioperative management in patients undergoing cardiovascular surgery, although there is controversy on the routine application of the PAC during cardiac surgery. In our hospital, the PAC is routinely placed by monitoring the pressure waveform after induction of general anesthesia. We are usually successful in placing the PAC within a minute [1], but in some cases, we failed or needed longer time.

One of the major reasons why the placement becomes difficult is because there are obstacles such as a history of tricuspid ring annuloplasty, pulmonary artery stenosis, and the intravenous pacemaker lead (PML) in its placement route. However, with the exception of some case reports [2–4], few publications have examined the difficulty of PAC placement in patients with these factors. Therefore, we previously measured the PAC placement time in patients with a PML [5] and found that the PAC placement time did not significantly change with or without a PML, suggesting that a PML did not significantly affect PAC placement.

The tricuspid valve is an atrioventricular valve consisting of three lobes, anterior leaflet, posterior leaflet, and septal leaflet. Previous studies showed that device leads, such as pacemaker leads, were located at the tricuspid valve annulus with three-dimensional (3D) transthoracic echocardiography [6, 7] and leads were frequently located between posterior and septal leaflets [7].

Based on these findings, we hypothesized that a PML does not interfere with the PAC placement because the PML and the PAC pass through different position at the level of the tricuspid annulus. In this study, we used the 3D transesophageal echocardiography (TEE) to visualize the position of the catheter position at the tricuspid annulus to examine where the PAC passes through at the level of the tricuspid annulus. Furthermore, we investigated possibility that the location of PAC at the level of the tricuspid annulus may affect the time required for its placement.

## Methods

This study was approved by the institutional review board (No.19-22), and informed consent was obtained from all eligible patients. It was registered in the UMIN Clinical Trial Registry (UMIN 000036040). This study was conducted from January to August 2019 at the Sakurabashi-Watanabe Hospital, Osaka, Japan. We prospectively examined the PAC location at the level of the tricuspid annulus in 135 consecutive adult patients undergoing cardiovascular surgery. We excluded cases whose PAC was placed before surgery, whose 3D TEE view was not clear, whose PAC had to be placed through the left internal jugular vein, whose data had a missing value, and those who had a history of tricuspid annuloplasty.

The electrocardiogram, invasive arterial blood pressure, oxygen saturation, and endo-tidal carbon dioxide were monitored on all patients. After induction of general anesthesia with midazolam of 0.1mg/kg, fentanyl of 200*μ*g, and vecuronium of 0.1mg/kg, mechanical ventilation was started following tracheal intubation. Anesthesia was maintained with propofol or sevoflurane combined with remifentanil and fentanyl. The PAC (continuous cardiac output/SvO2 Catheter 744HF75, Edwards Lifescience, Irvine, CA, USA) was inserted through the right internal jugular vein and placed by one of the three staff anesthesiologists in our hospital. First, the introducer sheath was placed through the right internal jugular vein and the PAC was started floating through the sheath until the pressure waveform changed to the central venous pressure pattern. Then, the balloon was inflated with 1.5 ml of air. With inflated balloon, the catheter was floated into the pulmonary artery. Once the waveform of the pulmonary artery was first observed, we inserted the catheter approximately 2–3cm forward and deflated the balloon.

The time required for the placement of PAC was measured as follows. The right atrium time (RAT) and the right ventricle time (RVT) were defined as the duration of time required for the catheter to float from the CVP position through the tricuspid valve to the right ventricle and that from the right ventricle through the pulmonary valve to the pulmonary artery, respectively.

**Fig. 1:**
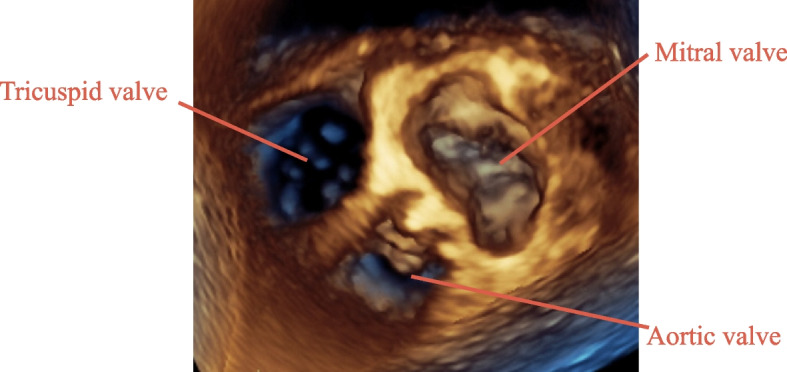
The 3-dimensional echocardiographic image of the tricuspid valve enface view from right atrium. The tricuspid valve is seen to the left, the mitral valve on the right, and the aortic valve below the two valves

The 3D TEE studies were performed using the Phillips iE90 ultrasound system (Philips Healthcare, Eindhovent, Netherlands). The image of the tricuspid valve was created using the mid-esophageal 4-chamber view in full volume 3D mode wherein the pyramidal scan volume could capture the whole tricuspid valve, mitral valve, and aortic valve (Fig. 1). This image makes it easier to determine the orientation of the tricuspid valve. After the gain was optimized, the image was cropped to visualize the tricuspid valve in the surgeon’s view from right atrium perspective. Originally, we planned to let the tricuspid valve be oriented with the septum in 6 o’clock position in accordance with the American Society of Echocardiography guideline [8], but we found that the images were easier to understand using a view that depicted the three valves all together as we are accustomed to use.

**Fig. 2:**
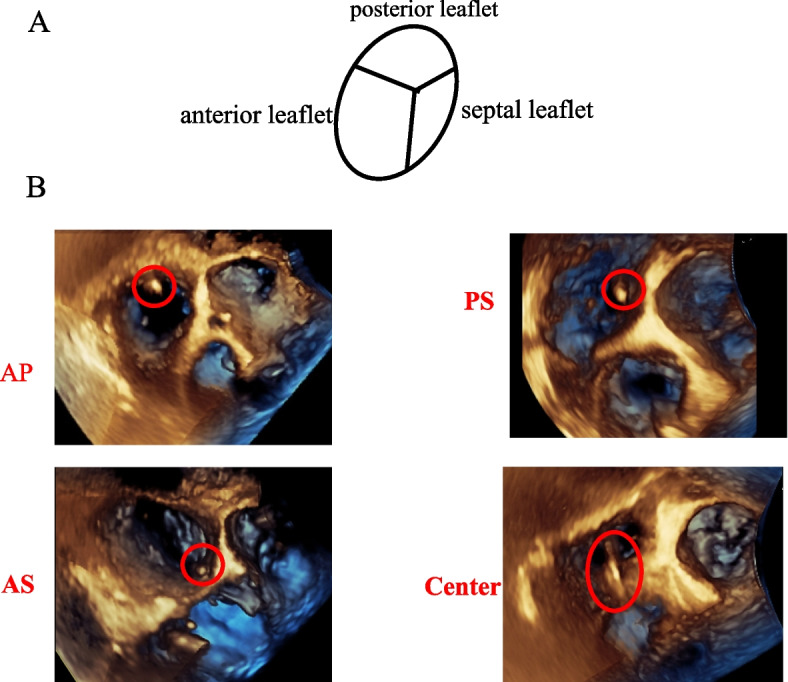
Schematic view of the tricuspid valve with leaflets **A** and pulmonary artery catheter location on 3D transesophageal echocardiography **B**. Example of four categories of the catheter locations at the tricuspid valve annulus. AP anterior-posterior leaflets, SP septal-posterior leaflets, AS anterior-septal leaflets, center center of the three leaflets

The tricuspid valve has three leaflets, anterior, posterior, and septal. The anterior leaflet is the largest and covers the infundibulum anteriorly to the inferolateral wall posteriorly. The posterior leaflet has many variations because of the multiple scallops. The septal leaflet is the smallest and arises directly from the annulus above the interventricular septum [9]. We divided the PAC position at the tricuspid annulus into four categories (Fig. 2). If it was in the commissure, the position was defined according to the commissural location between the respective two tricuspid leaflets, antero-posterior leaflets (AP), antero-septal leaflets (AS), and septal-posterior leaflets (SP), respectively. Everything else was defined as the center. After the PAC was placed, TEE probe was inserted. We acquired the 3D image of tricuspid valve and determined the PAC position before the surgery was started.

### Sample size calculation and statistics

Based on our previous study [1], we planned to collect 100 cases and then calculate the sample size. The sample size was calculated with 80 % power (*a*=0.05 and *b*=0.20) to detect the mean difference of RVT between the AP position and the SP position, and it was calculated that 128 patients would be necessary to demonstrate a significant difference.

Data were expressed as mean ± SD or as a median and interquartile range as appropriate. The preoperative patient data were analyzed by the analysis of variance or Kruskal-Wallis test as appropriate. The placement time was analyzed by Kruskal-Wallis test followed by Mann-Whitney test with Holm multiple comparisons to specify differences between groups. The statistical analysis was performed by SPSS (IBM Corporation, USA) version 20.0. *P* < 0.05 was considered statistically significant.

## Results

**Fig. 3:**
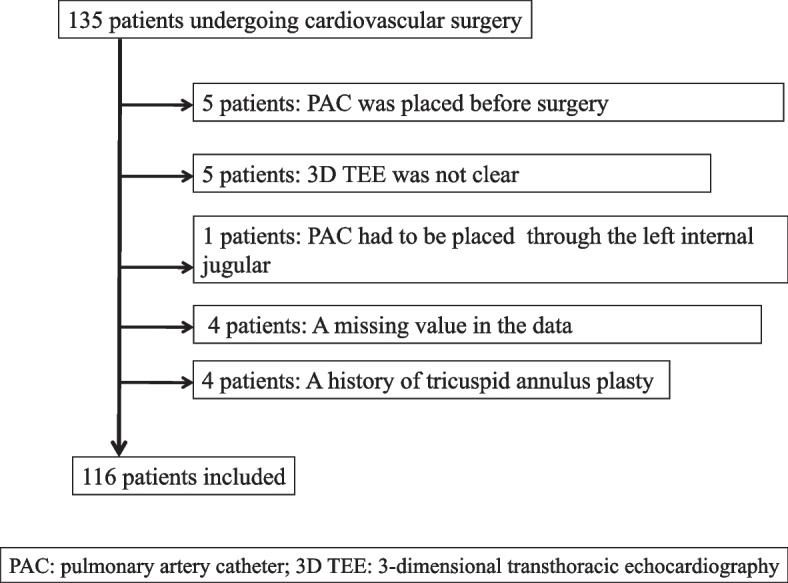
Patient inclusions and exclusions

Nineteen patients gave consent but were not included in the study for the following reasons: PAC was placed before surgery in five patients, 3D TEE view was not clear in four patients, PAC had to be placed through the left internal jugular vein in one patient, four patients had a missing value in the data, and four patients had a history of tricuspid annuloplasty. The remaining 116 patients were included (75 males, 41 females) for analysis (Fig. 3).

The patient’s demographic data including the patient’s surgical diseases is presented in Table 1. Table 2 shows the number and percentage of each PAC location in the tricuspid orifice. Of the 116 cases, 78 cases were positioned at the AP (67.2 %), 25 cases were positioned at the SP (21.6 %), 2 cases were positioned at the AS (1.7 %), and 11 cases were positioned at the center (9.5 %), respectively. Thus, a PAC passes through the tricuspid valve at the AP most frequently. Table 2 also shows the comparison of PAC placement time among the PAC positions at the tricuspid annulus. We found a significant difference in RVT, although RAT did not reach statistical significance. The following multiple comparison showed that there was significant difference between the AP and the SP and between the center and the SP.

**Table 1:** Patient characteristics

Age (year)	71 ± 10
Sex (male/female)	75/41
Height (cm)	160 ± 11
Weight (kg)	60 ± 13
CTR (%)	52 ± 7
LVEF (%)	60 ± 14
Degree of TR	1 (1–2)
Disease	
AS (19), AR (13), MR (29), TR (4), CAD (12), PVF (9), AAE (2), CAD + AS (3), CAD+AR (2), CAD + MR (1), AR + MR (3), AS + MR(3), MR + TR (2), AAE + MR (1), TAA + MR (1), TAA + AR (3), VSP (3), and Myxoma (1)	

Data were expressed as means ± SD or as a median range and interquartile range as appropriate (*n* = 116)

*CTR* cardiothoracic ratio, *LVEF* left ventricular ejection fraction, *TR* tricuspid regurgitation, *AS* aortic stenosis, *AR* aortic regurgitation, *MR* mitral regurgitation, *CAD* coronary artery disease, *PVF* prosthetic valve failure, *AAE* annuloaortic ectasia, *TAA* thoracic aorta aneurysm, *VSP* ventricular septal perforation

**Table 2:** PAC location and PAC placement time

Location	Anterior-septal leaflets	Septal-posterior leaflets	Anterior-septal leaflets	Center	*P* value
*N* (frequency)	78 (67.2 %)	25 (21.6 %)	2 (1.8 %)	11 (9.5 %)	
Right atrium time (s)	7 [5–11]	7 [5–25]	16.5 [15.25–17.75]	6 [6–9]	0.222
Right ventricle time (s)	10* [7–15]	15 [9.5–33]	15 [15–15]	8* [6–12]	0.016

Each time is expressed in the median and interquartile range

*P <0.05 vs septal-posterior leaflets

*PAC* pulmonary artery catheter, *Right atrium time*, duration of time required for the catheter to float from the CVP position through the tricuspid valve to the right ventricle; *Right ventricle time*, duration of time required for the catheter to float from the right ventricle through the pulmonary valve to the pulmonary artery

## Discussion

The principal finding of this study is that 3D TEE is available to identify the PAC location at the level of tricuspid annulus and about 70 % of the catheter positioned between the AP. Furthermore, the PAC placement time was significantly different depending on the PAC positions at the tricuspid annulus.

**Fig. 4:**
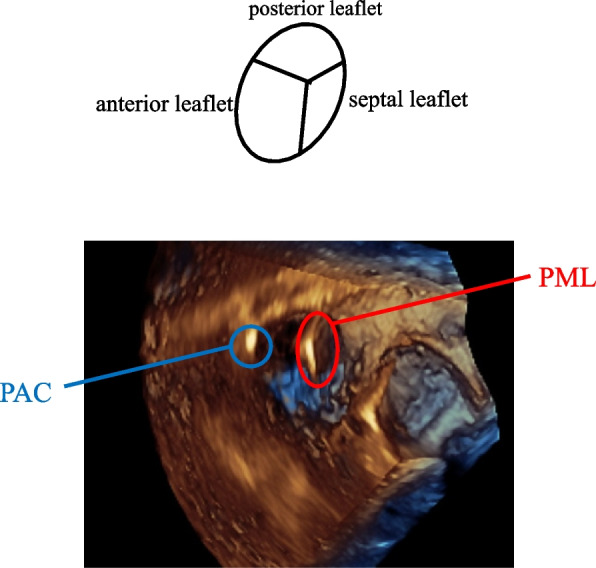
The 3-dimensional echocardiographic image of tricuspid valve with both pacemaker lead (red circle) and pulmonary artery catheter (blue circle). The pacemaker lead was positioned between the septal-posterior leaflets, and a pulmonary artery catheter was between the anterior-posterior leaflets, respectively. PML, pacemaker lead, PAC pulmonary artery catheter

Previous studies with 3D TTE showed that device leads including PML predominantly positioned between the SP [7]. On the contrary, the present study documents that a PAC passes through the tricuspid valve at the AP most frequently. Thus, a PML and a PAC may be positioned at different commissure in the tricuspid annulus and these data may justify our hypothesis that a PML does not interfere with PAC placement. Here, we would like to present a typical 3D tricuspid valve image of one patient in whom both a PML and a PAC were placed in Fig. 4, showing that the PML was firmly attached with the SP commissure and the PAC was positioned between the AP.

To our knowledge, this is the first report to examine the commissural location of the tricuspid valve where the catheter passes. We also showed that the PAC position in the tricuspid valve might be a factor for a smooth placement of the PAC. Our study showed that about 90 % of the PAC were positioned at the AP or the SP of the tricuspid annulus and that the RVT was significantly shorter in cases where the PAC was positioned in the AP. This finding could be explained by the anatomical position of the three tricuspid valve leaflets, that is, the AP being located on the outermost side of the right ventricular inflow tract in the tricuspid annulus. Thus, if the PAC is positioned in the AP, the catheter goes through the least angled route and floats into the pulmonary artery, resulting in smoother placement. In addition, our data may have some clinical suggestions. When it is difficult for the PAC to float into the pulmonary artery, it might be possible that the catheter is stuck in SP. We recommend pulling the catheter out to the right atrium and starting over again just before the tricuspid valve.

Here, we would like to speculate why a PML and a PAC tend to pass through different sites of tricuspid valve. A PML is hard and the tip of PML is placed in the right ventricle, not the pulmonary artery, so the lead may be placed through the shortest route, which includes posterior-septal leaflets of the tricuspid valve. On the other hand, a PAC is soft and must be placed into the pulmonary artery. As described above, location of AP is convenient for a PAC to go through the least angled route and floats into the pulmonary artery.

While fluoroscopic guidance is commonly used to place the PAC, tricuspid valve leaflets are not seen on fluoroscopy. Two-dimensional (2D) TEE is also commonly used in the operation room evaluate where to place the PAC. However, only two leaflets can be displayed simultaneously in any 2D image plane. On the other hand, 3D TEE is considered useful because it can construct an enface view when we evaluate the morphology of the tricuspid valve, which may help in smoother PAC placement.

There were some limitations in this study. First, we examined the location after the catheter placement, but not at the time of insertion. Thus, one may deduced that there is a possibility that the location may shift after the placement. We have to acknowledge this possibility, since we have no definite data whether the location shift may occur after the placement. Second, the number of the AS and the center samples were fewer compared to that of the AP and SP samples due to our study design. Further study accumulating the number of the AS and the center is needed to evaluate the effect of these positions, and we acknowledge the possibility that accumulation of further number of subjects could change our conclusion in the future. Third, our result on the PAC placement between the AP and the SP was dependent on the statistical analysis, so the clinical significance of our data would be interpreted with caution. Fourth, we placed the PAC by conventional monitoring of the pressure waveform. However, now, visual guidance such as TEE and fluoroscopy may be used for the PAC placement. We acknowledge that it is not clear whether the present results may be applicable to the placement with these visualizations.

In conclusion, this study confirms that 3D TEE is available to identify the PAC location at the level of the tricuspid annulus and almost 70 % of the catheter positioned between the anterior and posterior leaflets. Furthermore, the PAC placement time was significantly longer, when the catheter was positioned between the septal and posterior leaflets.

## Data Availability

The data that support the findings of this study are available from the corresponding author on reasonable reason.

## Abbreviations

PAC: Pulmonary artery catheter
PML: Pacemaker lead
3D: 3-Dimensional
TEE: Transesophageal echocardiography
RAT: Right atrium time
RVT: Right ventricle time
AP: Antero-posterior leaflets
AS: Antero-septal leaflets
SP: Septal-posterior leaflets
